# Serotonin Reuptake Inhibitors and the Gut Microbiome: Significance of the Gut Microbiome in Relation to Mechanism of Action, Treatment Response, Side Effects, and Tachyphylaxis

**DOI:** 10.3389/fpsyt.2021.682868

**Published:** 2021-05-26

**Authors:** Peter Sjöstedt, Jesper Enander, Josef Isung

**Affiliations:** ^1^Capio Proximity, Ramsay Santé, Gothenburg, Sweden; ^2^Centre for Psychiatry Research, Department of Clinical Neuroscience, Karolinska Institute, Stockholm Health Care Services, Region Stockholm, Stockholm, Sweden

**Keywords:** selective serotonergic reuptake inhibitors, psychopharmacology, gut brain axis, monoamine hypothesis, microbiome and dysbiosis

## Abstract

The monoamine hypothesis of psychopharmacology has been dominating the biological psychiatric research field for decades. Currently psychiatric research has increasingly appreciated psychiatric disorders and suicidal behavior as being highly complex and multi-etiological. In this pathway the gut microbiome and its interrelationship with the brain is gaining traction. The usage of selective serotonin reuptake inhibitors (SSRIs) is increasing in the general population. This is due to their effect on a broad range of psychiatric disorders, and their favorable side effect profile. Still, there are enigmatic aspects about SSRIs, such as the difficulty to predict effect in individual patients, inter-individual differences in side effect, tachyphylaxis (a sudden loss of response to a certain drug), and to date, uncertainties on how they exert their clinical effect. A majority of the serotonin in the human body is produced within the gut, and SSRIs affect enteric neurons. They also exhibit antimicrobial properties that comes with the potential of disrupting microbial hemostasis. We propose that the role of the gut-brain axis and the gut microbiome in relation to psychopharmacology should be more highlighted. With this article, together with similar articles, we would like to provide a hypothetical framework for future studies within this field. We believe that this would have the potential to provide a paradigm shift within the field of psychopharmacology, and result in findings that potentially could contribute to the development of a more personalized and tailored treatment.

## Introduction

The connection between the gut and the mind, the so called gut-brain axis (1), is a burgeoning research field that holds promise to further our understanding of the pathophysiological disruptions underlying complex disorders, such as psychiatric disorders and suicidal behavior (1, 2). Even though this field is sometimes thought of as novel, history shows that the notion of a connection between the gut and the mind has been recurring within the medical science since ancient times (3, 4). This is reflected in our language, such as the expression “butterflies in the stomach” as a term of worry appraisal, and the term having a “gut feeling” (5). Indeed, the term “hypochondriasis” (6), literally meaning below the ribs, implicates that the root of this disorder is due to imbalances in the stomach (6). During the eighteenth and up until the early twentieth century a dominant theory on behavioral disorders was that imbalances in eating behavior and a sedentary lifestyle had an impact on the gut, thought to explain the “epidemic” of dyspepsia (also known as indigestion; a symptom of discomfort and pain from the upper gastrointestinal tract) in society at that time. Dyspepsia was thought of as the root cause of psychiatric ill health, and even suicide (3). This reductionistic way of thinking changed its route during the twentieth century, when an opposed reductionistic direction of thinking instead stated that stress and anxiety was the root cause of stomach illnesses, such as peptic ulcers, as well as certain other somatic disorders (3). The current views of a bidirectionality between the gut and the brain—and the view of the body as a whole, rather than a dualistic construct between “body and mind” (1, 4)—arguably gives a more biologically rational way of thinking about pathophysiological complex disruptions that lead to psychiatric disorders, as well as other complex conditions. Indeed, our current view on these disorders as systemic, rather than solely being intrinsic disorders of the brain, seems more plausible considering known genetic links and associations between several psychiatric disorders and e.g., autoimmune diseases, primary immunodeficiencies, and chronic infections (1, 7–13).

## Aims

In this opinion article we would like to propose alternate views on selective serotonin reuptake inhibitors (SSRIs) in the treatment of psychiatric disorders, relating them to the gut-brain axis and the gut microbiome. First, we give a brief overview on serotonin and its role in the gut. We then expand on the plausible gut microbial involvement in the mechanisms of SSRI-treatment effect, treatment response, side effects, and tachyphylaxis (i.e., a sudden loss of therapeutic response upon an initiated or a repeated drug use). A few pre-clinical studies that are cited have provided some preliminary evidence, supporting the involvement of the gut microbiome with psychopharmacological side effects (14), and even in the mechanism of action (15). A narrative review have proposed similar arguments as we do, however in a broader perspective, focusing on all psychopharmacological compounds (16). A similar argument has been proposed for other psychopharmacological compounds as well, but due to the fact that such a high degree of the serotonin synthesis takes place in the gut (17), and to the widespread usage of SSRIs (18), we wanted to focus on this specific class of drugs as we believe that this would be of particular interest. This article is not suggestive of being an exhaustive review, but rather an attempt to propose an integrative hypothesis on this topic and give “food for thought.”

## The Monoamine Hypothesis

The monoamine hypothesis of depression and other psychiatric disorders has been dominant for decades (19). The hypothesis in relation to affective disorders was proposed due to converging evidence from different studies, such as (a) the induction of depression shown in some individuals, and also depressive-like behavior in animals, as a side effect to the use of the monoamine depleting compound called reserpine, and (b) the antidepressant effect that was evident using compounds such as monoamine oxidase inhibitors (MAOi) and tricyclic antidepressants (TCA) that raised the monoamine levels in the brain (19). In recent years the implicated pathophysiological importance of this hypothesis has been disputed (19). This is partly due to the obviously insufficient treatment effect for many patients of the different compounds that target the monoamines, as well as the emergence of new treatments with antidepressant effect targeting other transmitters such as recently the glutamatergic system, most noteworthy being studies using ketamine (19). Recent hypotheses for mood disorders as well as suicidal behavior have been focusing on several other proposed pathophysiological mechanisms such as neuroinflammation, immune dysregulation, disruptions in neurogenesis, imbalances in neuropeptides and growth factors, and also expanding on other types of neurotransmitters (19–23). In this shift and broadening of perspectives an imbalance in cerebral networks are highlighted, where the monoamine system is appreciated as an integral part in network signaling and modulation (19). From this perspective disruptions in the monoamine system is thought of as one manifestation among the many heterogeneous disruptions that would form the basis of an underlying pathophysiology (19).

## History Of Selective Serotonin Reuptake Inhibitors

Since SSRIs were first introduced in clinical practice about 30 years ago, the world has seen an immense increase in their use (19). Treatment is now extremely common within the general population, with estimates that up to 13% of the population in the US are prevalent users (18). Long-term positive effects on a population level, in treating the global epidemic of disability due to psychiatric disorders, arguably is yet to be seen (24, 25). With the exception of suicide rates (26), literature rather point in an opposite direction, showing an increased burden of disability due to psychiatric problems in the general population (24, 27). Obviously, the increased burden of psychiatric disorders does not necessarily imply that this is due to the lack of efficacy from antidepressants, since multiple other factors may be more influential (25). Many patients evidently have experienced help from the use of SSRIs, both for depression (28), and for anxiety disorders (29, 30). This is especially the case for patients with obsessive-compulsive disorder, where serotonergic agents seem to be more specific than non-serotonergic agents (31). A clinical challenge in relation to SSRI treatment is that many patients experience treatment-resistance in spite of using SSRIs at a therapeutic level and during a sufficient time-frame (32). Moreover, some patients experience intolerable side effects (28), and a significant proportion of patients experience the more enigmatic case of tachyphylaxis (“poop-out effect”), i.e., that the treatment effect suddenly subsides after an initially reported beneficial effect (33). Tachyphylaxis is also reported to be associated with the development of a more general treatment-resistant condition, with lower odds of a subsequent efficacious treatment with the same or other types of drugs (33). Another enigmatic aspect of SSRIs is that the occupancy of the serotonin transporter (SERT) reaches on average 80% already within the lower therapeutic dose-ranges (34, 35). In spite of this, some patients need higher doses to experience symptom relief or remission (36). This fact speaks against SERT inhibition being the sole mechanism of action, given the high SERT occupancy already at the lower dose intervals, and that increasing doses do not lead to a linear increase in occupancy, but reaches a plateau (34).

## The Gut Microbiome

In recent years the significance of our microbiome has been widely highlighted, and often viewed upon with hope of finding new ways of understanding complex disorders (7, 37). Especially the commensal microbes of the gut, i.e., the gut microbiome, has been of interest since there is evidence of cross-talk between the gut and the brain (1, 4). Microbial dysbiosis, i.e., imbalances within the gut microbiome, is thought to be one variable that could be of interest in the pathophysiological processes underlying psychiatric disorders and suicidal behavior (2, 7). Previous estimates have usually stated that the gut contains 10-fold more microbes than the amount of cells that makes up the human body, however updated estimates rather suggests an ~1:1 cell ratio (38). Notwithstanding that the amount of cells may be approximately equal, the amount of unique genes within the gut microbiome is previously considered to surpass the human genome up to 250–800-fold (39). More recent estimates even suggests that this figure may be vastly underestimated as the complexity of the gut microbiome is yet to be fully unraveled (39, 40). In sum, the complexity of this colony motivates the recent interest seen in this research field. The colonization of the commensal microbes that constitutes the gut microbiome is a complex process that is generally thought to start at birth, but it could be a process taking place already *in utero* (41). Initial different compositions of the microbiome may depend on the mode of delivery (vaginal vs. caesarian section), whether the child is breastfed, skin-to-skin contact, different types of diet, etc. A dynamic process then proceeds toward a gradual stabilization of the gut microbiome during adulthood (42). While the host genome is not easily manipulated, different dietary regimes are known to provide ways of manipulating the more plastic microbiome. In terms of treatment interventions, this is mainly discussed in the context of pre- or probiotic regimens. Prebiotics mainly refers to different types of indigestible fibers that may ferment in the colon, and act as substrate and stimulate the growth of a beneficial microbiome that colonize the colon, whereas probiotics are the dietary supplementation with microbes that should replenish the gut with more health beneficial strains (43). This type of manipulation could provide an opportunity to intervene as prevention of disorders or as treatment of manifest disorders, also in the context of neuropsychiatric disorders (43, 44). The problem with this avenue of interventions is the vast complexity, where manipulations may have both beneficial and detrimental effects at the same time depending on the different strains affected in relation to the targeted disorder (43). Other potential ways that one might manipulate or restore (e.g., after treatment with antibiotics) the gut microbiome is through fecal microbiota transplants (FMT). This has proven to be a highly effective way of treating a type of severely disabling chronic diarrhea due to colonization of clostridium difficile (45). It has also been evaluated in pre-clinical as well as a few small clinical trials in relation to neuropsychiatric symptoms (42). However, this is a burgeoning field and more research is warranted in regards to what precisely constitutes a healthy gut microbiome. Such research may lay the foundation for personalized medicine, where tailored FMT interventions could be used in the treatment of neuropsychiatric disorders. Relating to our emergent understanding of the gut microbiome, the innovation of new high-throughput sequencing techniques (39), allowing both cheaper and quicker sequencing of huge amounts of genetic code, has made the expansion and rapid development of this research field possible (39).

## Evidence of a Gut-Brain Effect

The discovery of the direct and fast pathways between the gut and the brain *via* the vagal nerve, and the indirect communication pathways through different modes of signaling (immune mediated, short chain fatty acid communication, synthesis and metabolism of monoamines etc.), in parallel with an increased understanding of the biological function and complexity of the gut microbiome, became catalysts for new, paradigm shifting ideas—with implications even for how we understand “the self” to a certain extent (46). The biological understanding of “the self” has hitherto relied upon the stability of the human genome, the dynamics of the immune system, neurodevelopment, and the cognitive processes that emanate from the brain's function (46). In the light of the discussed findings it is evident that what constitutes and influences our “self” is actually, in addition, composed of colonies of microbes, vastly outnumbering the host in terms of genetic code (46). The gut microbiome has important physiological functions for our immune system, for emotional processes and cognitive functioning, and even for the metabolism of drugs and neurotransmitters, as well as having a direct production of neurotransmitters and neuropeptides (1, 7, 46). To date, our knowledge of how the gut microbiome may have an impact on health-related outcomes relates mainly to its involvement in regulating the metabolism of nutrients and to how differential individual compositions of bacterial strains are implicated in the risk of obesity (39). Differential microbiome compositions are even suggestive of an increased risk for diabetes and atherosclerosis, through the induction of inflammation (39). Pre-clinical studies suggest that the composition of the microbiome is disrupted by states of depression and anxiety, but also that this is a bidirectional effect (4). In essence, the microbiome may affect the induction of depressive or anxious states, and the induced state may lead to aggravated disruptions, through increased cortisol levels and immune dysregulation, giving rise to a circulus vitiosus (4). Additionally, patients with mood disorders are strikingly often affected by irritable bowel syndrome (IBS), which is reported to be seen in nearly half of all mood disorder patients (4). IBS is implicated to be due to a microbial dysbiosis (47), and the use of SSRIs is common as a pharmaceutical strategy to alleviate IBS symptoms (4). There are also studies that suggest the importance of the gut microbiome directly in the metabolism of psychotropic drugs, such as certain benzodiazepines and antipsychotics (4). Finally, more direct evidence in relation to side effects is proposed from two pre-clinical studies, looking at the common antipsychotic compound olanzapine (14, 48). Olanzapine is known for its proneness to induce severe weight gain and metabolic disruptions (14). Olanzapine has been shown to interact with the gut microbiome, where the resulting dysbiosis seemed to mediate the weight gain, i.e., it seemed to depend upon the presence of a gut microbiome (14). In a follow-up study the same researchers could confirm that the weight gain could be alleviated by adding antibiotics eradicating the gut microbiome (48). Relating to our topic, the gut could be considered to be of particular interest in relation to SSRIs, considering that 95% of the serotonin production in the body takes place in the gut, mainly by the intestinal enterochromaffin cells (5). Serotonin is the main neurotransmitter used by enteric neurons in the regulation of gut motility (5). Interestingly, there is also evidence of serotonin production and turnover by the gut microbiome itself (5).

## Psychotropics Acting as Antibiotics

It is interesting that, beside the direct actions that SSRIs have on the gastrointestinal tract with its abundance of serotonergic neurons (49), SSRIs (as well as several other psychotropic drugs) exert antibiotic effects, which may have direct consequences in disrupting the integrity and stability of the gut microbiome (50, 51). One of the very first antidepressant drug discoveries was the anti-tuberculotic agent iproniazid that functions as an inhibitor of MAO-A, thus inhibiting the enzymatic degradation of serotonin, noradrenalin and dopamine. There is also ongoing research regarding the possible antidepressant effect from common antibiotic drugs such as tetracyclines. The use of antibiotics can disrupt gut microbiome homeostasis and induce microbial dysbiosis (16). The gut microbiome could also be affected in terms of antimicrobial resistance through the utilization of developed “resistance genes” (52). The “resistance genes” that protect the bacteria from antibiotics are collectively called the “resistome” (52). Previous evidence has shown that the gut microbiome is quite resilient in its recovery after the use of antibiotics. However, a positive selection of bacteria with “resistance genes” is shown to be a consequence of antibiotic usage, which may introduce a dysbiosis through preferential cloning depending on the type of bacteria that may have differentially achieved a protective “resistome” (52). Furthermore, specifically in the context of SSRIs as reviewed by McGovern et al. (53), antimicrobial properties are described for all SSRIs in different proportions, where sertraline, fluoxetine and paroxetine, in that order, seemed to affect more strains and have the strongest antimicrobial effect, followed by fluvoxamine, escitalopram and citalopram being the SSRIs having the least impact. From different studies, it could also be estimated that sertraline, fluoxetine, paroxetine and fluvoxamine remain in the ileum and colon in concentrations high enough and for enough time (calculated in relation to their minimal inhibitory concentration for different microbes) to have a direct influence on the gut microbiome of both the colon and the small intestine (54). Since SSRIs also seem to affect microbial defense systems, such as efflux pumps protecting microbes from antibiotics and other drugs with antimicrobial activities, SSRIs might act in synergy with drugs affecting different strains and thus induce a microbial dysbiosis. This is arguably of special importance in relation to SSRIs, given the more chronic type of administration that is usually considered when treating patients with these types of drugs, and thus adds additional complexity to plausible interaction effects together with concomitant drug use. As previously discussed, evidence suggest that the gut microbiome can have significant effects on emotions, behaviors, metabolic risks, and metabolism of drugs (4), and thus may be involved in the pathomechanisms precipitating psychiatric disorders and suicidal behavior (2, 4). Thus, the effect on emotional responses and behavior, as well as side effects, from the chronic use of SSRIs (and other psychotropic drugs) could be due to their antimicrobial properties and long-term effect on the microbial composition.

## The Gut Microbiome and Treatment Effect From SSRI

SSRIs are currently thought to exert their effects against mood and anxiety disorders mainly through SERT-inhibition in the brain and secondary effects on post-synaptic serotonin receptors, and further downstream on growth factors and neuropeptides (15, 19). To date, however, there still exists a controversy relating to how SSRI works, not least since there are many enigmatic aspects on e.g., treatment-resistance in a significant number of individuals, and different side effect profiles. As discussed above, it would be reasonable to speculate that the gut microbiome may be indirectly, or plausibly even directly involved in the mechanism of action from SSRIs on psychiatric disorders. In line with this, a pre-clinical trial has provided evidence that five different antidepressants (including two SSRIs) can affect both the balance and the integrity of the gut microbiome, and that both treatment effect and side effects could be modulated by the replenishment of different strains of probiotics (15). This is highly interesting, and hopefully the first of many similar studies to follow, looking at possible mechanisms underlying such associations. Beside from the issues relating to treatment-resistance seen in a high proportion of patients, this might also give a further understanding on other enigmatic aspects, e.g., that some patients need higher doses to experience treatment response even though this does not lead to significantly higher SERT bindning, as discussed above (19). Whether this is due to the gut microbiomes inter-individual effect on drug metabolism (55), to gut microbes affecting the brain *via* the gut-brain axis or through other enteroendocrine signaling pathways, e.g., microbe production of neurotransmitters and neuropeptides, are questions that warrants further studies.

## The Gut Microbiome and Side Effects From SSRI

The fact that there are inter-individual differences in side effects could arguably be explained by gut microbial dysbiosis. The fact that enteric serotonergic neurons are involved in the regulation of gut motility (49) can readily explain adverse effects such as nausea, constipation or diarrhea (15). But the formerly mentioned studies referring to weight gain induction from olanzapine (14) could perhaps also be extrapolated to SSRIs. Recent evidence has been provided from a small pre-clinical study looking specifically at fluoxetine in rats, where weight gain was accompanied by induction of the disruption of certain bacterial strains such as lactobacilli, which are known to be involved in the regulation of body weight (56). It is well-known both from clinical trials and as a clinical experience that some patients seem to be extra vulnerable and can experience significant weight gain from the use of SSRIs (57). Whether this is due to their constitutional gut microbiome composition would be an interesting area of research. This could have direct clinical implications if it would result in means to attenuate these side effects using pre- and/or probiotic compounds (43) or by other ways of manipulating the microbiome, such as plausibly through the use of FMT (42). If this understanding could be furthered to other common SSRI related side effects as well is also in need of further exploration.

## The Case of Tachyphylaxis

The final question that we would like to discuss is the phenomenon that some patients on previously effective treatment develop a so called “poop-out” effect, i.e., evidence of tachyphylaxis (33, 58). This phenomenon has been known for decades, and is reported to occur in about 25% of patients treated with SSRIs for depression. It is also reported that previous long-term treatment with SSRIs may affect prospective treatment periods negatively for some patients, resulting in less beneficial effects compared to the first treatment course. The causes for these inter-individual differences still remains a mystery (33, 58). The apparent effects of SSRIs, among other drugs, acting as antimicrobials would perhaps be one variable to take into consideration. The treatment for mood- and anxiety disorders generally occur over months and even years. Chronic administration of a compound with known antimicrobial properties could plausibly have enduring effects on the fine-tuned balance, integrity and composition of the microbiome. This might be an effect that takes place through induced “resistance gene” selection. Thus, the phenomenon of tachyphylaxis could plausibly be explained by effects from the chronic administration of SSRIs on the composition of microbes through its antimicrobial effect and a resultant dysbiosis (16). This could be a plausible explanation for why tachyphylaxis seems to be associated with treatment-resistance also against subsequent treatment trials (16). In this line of research, it would be interesting to explore not only if these disruptions occur, but also what such a “microbial fingerprint” would look like. This warrants longitudinal study designs to learn if such disruptions eventually dissipate or if there are means to manipulate them in order to restore balance. This could perhaps be achieved through pre- and/or probiotic compounds, fecal microbiota transplants or through other innovative and novel strategies for manipulating and taking advantage of the dynamics of the gut microbiome.

## Discussion

We have described the previous more reductionistic view on the underlying pathophysiological disruptions of psychiatric conditions as either solely emanating from the gut or, in later years, as merely intrinsic “brain disorders.” Today we probably have a more systemic view on the complex interactions, the fine-tuning and the contingent development of the gut microbiome, the immune system and the development of the central nervous system, not only for psychiatric disorders but also for other complex disorders such as autoimmune diseases. The shared disruptions and patterns of comorbidity between psychiatric disorders, inflammation, and autoimmune diseases strengthens this view (8, 9, 12, 59). Adding this perspective could challenge long-lived beliefs such as the monoamine hypothesis, and other propensities toward simplified explanations of complex psychiatric disorders (60), and help moving the field forward in relation to enigmatic issues surrounding treatment-resistance, patterns of side effects, and tachyphylaxis. We hope that this opinion piece will encourage more pre-clinical and, importantly, clinical studies in naturalistic settings, examining these associations. It would be feasible initially to longitudinally sample individuals that are being treated with SSRIs in clinical settings and follow them in relation to treatment response, side effects and tachyphylaxis, and relate this to their bacterial composition at baseline and at follow-up after exposure to SSRI.

## Clinical Perspectives and Implications

A better understanding of the microbiome-drug interaction would be of substantial importance. Evidence of dysbiosis as a consequence of treatment, or unbeneficial “fingerprints” of gut microbes in relation to treatment, or even therapeutic predictive abilities from the gut microbiome composition, both regarding treatment response and relating to side effects and tachyphylaxis, has the potential to inspire a new avenue of research that could provide better and personalized treatments, through an advanced understanding of the dynamic gut microbiome. This could, for instance, lead to plausible add-on treatments with beneficial pre- and/or probiotics or even FMT, that could optimize outcomes relating to treatment response, side effects, and bring new insights as well as possible treatments and cures for tachyphylaxis.

## Data Availability Statement

The original contributions presented in the study are included in the article/supplementary material, further inquiries can be directed to the corresponding author.

## Author Contributions

All authors contributed to the intellectual content, writing, and final approval of the manuscript.

## Conflict of Interest

The authors declare that the research was conducted in the absence of any commercial or financial relationships that could be construed as a potential conflict of interest.
